# Powassan Virus Encephalitis, Minnesota, USA

**DOI:** 10.3201/eid1904.121651

**Published:** 2013-04

**Authors:** David F. Neitzel, Ruth Lynfield, Kirk Smith

**Affiliations:** Minnesota Department of Health, St. Paul, Minnesota, USA

**Keywords:** Powassan virus, Minnesota, diagnostic tests, viruses, *Suggested citation for this article:* Neitzel DF, Lynfield R, Smith K. Powassan virus encephalitis, Minnesota, USA [letter]. Emerg Infect Dis [Internet]. 2013 Apr [date cited]. http://dx.doi.org/10.3201/eid1904.121651

**To the Editor:** Birge and Sonnesyn report the first death of a Minnesota resident caused by Powassan virus (POWV) (*1*). However, they provide an inaccurate description of several critical diagnostic and surveillance issues concerning POWV.

The 17 POWV infections detected in Minnesota residents from 2008 through 2011 (6 cases were identified through 2010, not 8 as reported by Birge and Sonnesyn) (Minnesota Department of Health [MDH], unpub. data) were found through enhanced surveillance. Health alerts to Minnesota medical providers described POWV as a possible etiologic agent for viral meningitis and encephalitis. Providers consulted with MDH on suspected cases and submitted serum and cerebrospinal fluid specimens to MDH. MDH conducted serologic testing for endemic arboviruses (including POWV) and reverse transcription PCR (RT-PCR) for flaviviruses and POWV. MDH would not have detected any POWV infections without enhanced surveillance. Limited field studies also identified POWV-infected ticks in 4 Minnesota counties (not 2 as reported [*1*]) (MDH, unpub. data).

Commercial laboratories do not provide testing for POWV, and only a few state health department laboratories and the Centers for Disease Control and Prevention offer testing. Serologic testing (enzyme immunoassay with plaque-reduction neutralization testing confirmation) is preferred (*2*) because POWV RT-PCRs are not validated, and the short viremic periods of flaviviruses limit their usefulness (*3*).

Few POWV infections are identified by lineage (prototype vs. deer tick virus); Minnesota’s first case in 2008 was identified as a deer tick virus infection, but the lineage was unknown for the other 16 cases. However, many case-patients had likely exposure to *Ixodes scapularis* ticks (blacklegged ticks), the tick species associated with deer tick virus transmission, and viruses from all POWV-positive tick pools were confirmed as deer tick virus by sequencing. The distribution of the 2 lineages in North America is poorly understood, and most cases likely go undetected without specific POWV surveillance efforts.
